# Notes and Comments

**Published:** 1900-10

**Authors:** 


### Notes and Comments.[1]

WHAT THE DENTAL DEGREE STANDS FOR.—From Dr. Truman's timely editorial in the July JOURNAL we reproduce the following paragraph to give it additional emphasis:

"Dentistry has no reason to beg for recognition. Such a demand is always an indication of partial culture. Dentistry has a position secured by long years of patient work. It has demonstrated that the oral cavity contains within its borders sufficient to occupy the best thought for a lifetime of those who practise therein. It has proved to the doctors of medicine that for uncounted centuries they have neglected the principal source of disease, and that this neglect, to their discredit, still continues. It has proved that the pathological conditions of this cavity hold intimate relations with the general organization, and are a source of continued infection. It has proved that it has been possible to lessen the terrors of old age and prolong the period of life, and to make that period one of comparative comfort; and, finally, it has forced the respect of the men who treated it in the past with contempt, and they have been obliged, however unwillingly, to believe that the time has come when the partial degrees in dentistry should be abandoned."

---

HEADACHES FROM ALVEOLAR FISTULAS.—In a paper contributed to the *Pacific Dental Gazette* Dr. A. C. Hart says,—

"Patients often present themselves suffering with, as they term it, nervous headaches which have entirely disappeared on the closing up of one or more of these fistulous tracts. The constant drain upon the system, together with the psychical effects upon some extremely sensitive and nervous patient, to whom this discharge of pus becomes a source of dread and apprehension of what

---

[1] The assistant editor solicits contributions for this department,—new methods, new remedies and formulas, or any short practical note which may prove of value to the practitioner or student. Address 1338 Walnut Street, Philadelphia.

may result if this opening is not closed, is of sufficient importance to emphasize the need of closing these openings."

---

What does it profit?—It is often urged, says Dr. W. C. Barrett, that modern investigation and discovery has availed man little, and that all our knowledge of the microscopical world has not materially benefited the human race. We are told that the demonstration by Miller that dental caries is due to bacterial agency has not prevented the decay of teeth, nor lessened the demand for the service of the dentist. But it has armed him with new weapons, and enabled him better to meet the ravages of disease. The dentist can save teeth better now than he could even a decade ago, and he who best comprehends the discoveries of Miller can best save teeth.

---

Should Formalin be used for Dental Purposes?—Dr. A. H. Peck, of Chicago, before the Illinois State Dental Society, said,—"Having seen a number of cases that have been treated by physicians with various per cent. solutions of formalin in which more or less sloughing of the soft parts has resulted,—one which I saw not long since in which as low as a two per cent. solution was used, in connection with which considerable sloughing resulted,—and also because of the very vivid recollections of my own personal experiences with it, I have come to the conclusion that we should get along without it in the treatment of diseased conditions about the mouth."

---

Another Case of Cocaine Poisoning.—Dr. A. W. Harlan, in writing upon the subject, says,—

"Recently while getting ready to set a crown on the root of a central incisor, we placed a mat of paper about the size of a copper cent on the gum, saturated with a four per cent. solution of hydrochlorate of cocaine, and in about five minutes the patient was poisoned. She became limp and was not conscious of anything for four hours. It was with difficulty that she was made to walk, and she talked incessantly and incoherently.

"She was given one nitrite of amyl pearl and was made to inhale stronger ammonia and was given five cups of strong black coffee. The quantity of the solution used was about four minims, some of which was undoubtedly swallowed. The recovery was sudden and complete after four hours of incessant labor. No after-effects except some distress in the stomach on account of the large quantity of coffee. The next day she did not remember anything that occurred from the time she sat down in the chair until she was taken to the train to go home."

---

"Changes in the Weather increases Patronage."—In an interesting paper read before the Northern Ohio Dental Society, and published in the *Ohio Dental Journal,* Dr. Florence M. Taylor says that a change in the weather frequently causes trouble with the teeth; in other words, increases patronage. People often come, the doctor says, with a history of getting their feet wet. There is something about catching cold that predisposes to or causes pericementitis. A tooth that the patient knows needs filling is neglected because it does not cause any inconvenience, until he or she is caught in a storm or exposed to a draught, and then there is trouble. It may be nothing more than a congestion that counter-irritation or blood-letting will relieve. But the usual history after these attacks is that they return unless the tooth receives proper attention, and sooner or later an abscess is formed.

---

Trichloracetic Acid for Fistula from Alveolar Abscess.—For the treatment of a chronic fistula Dr. J. S. Ashbrook, in the *Dental Brief,* suggests the use of trichloracetic acid. With cotton carefully placed around a Donaldson broach, saturated with the acid, he says you can reach the bottom, and in most cases the fistula disappears after two applications. In all cases he uses the pure acid, in small quantities. In forty-eight hours all trace of its action has gone, except the good results; the mucous membrane peeling off, as it were, and a new layer forming.

---

Unjust Criticism.—Dr. L. P. Bethel says editorially in the *Ohio Journal,* "This spirit of criticism is too common. Simply because one man's methods may differ from yours is not cause for criticism and condemnation, for it may bring the same results and be as much easier for him as your method is for you. We are inclined to think that much of this criticism comes from immature thought, and often some petty jealousy or dislike seems to give rise to it.

"Recently in a dental meeting one member said, 'Dentists are the greatest braggarts on earth, etc.' He means you, all of you. Is not this a strong statement? It is true that we have some who are given to boasting, but these men soon find their level. Where we have one of this kind we have hundreds who tower above self-praise; men whose qualifications and attainments stamp them as superior without telling the world of their ability. That man is looked upon as mediocre who is continually telling of his great accomplishments. Why, then, should this remark be thrust at the whole profession? Is it just?"

---

Artificial Caries.—"I have just succeeded," writes Dr. S. A. Hopkins, "in producing artificial caries with a pure culture of the bacillus mesentericus vulgatis. The media was one per cent. glucose bouillon, and the time was nine months. There are many other forms, probably, that will do the same, and it will now be much easier to work them out. I believe this is the first *authentic* instance where decay has been produced by pure cultures of mouth bacteria, and I feel that it is an important step in advance. There is no secret about it. I should only be glad to find others working in the same line."

---

Preparation of Steel for regulating Appliances.—Dr. Genese, in the *Ohio Dental Journal,* says, after the steel appliance has been given the desired shape, it should be steeped in chloride of zinc, and then in pure molten tin. No oxidation will then take place; its tension is improved, and it can be united to any other metal by pure tin, using the chloride of zinc as a flux. If embedded in vulcanite it will not cause disintegration.

